# Bezafibrate Exerts Neuroprotective Effects in a Rat Model of Sporadic Alzheimer’s Disease

**DOI:** 10.3390/ph15020109

**Published:** 2022-01-18

**Authors:** Li-Fan Lin, Yun-Ting Jhao, Chuang-Hsin Chiu, Lu-Han Sun, Ta-Kai Chou, Chyng-Yann Shiue, Cheng-Yi Cheng, Kuo-Hsing Ma

**Affiliations:** 1 Graduate Institute of Medical Sciences, National Defense Medical Center, Taipei 114, Taiwan; fanlin2@gmail.com; 2 Department of Nuclear Medicine, Tri-Service General Hospital and National Defense Medical Center, Taipei 114, Taiwan; treasure316@gmail.com (C.-H.C.); dakaichou@gmail.com (T.-K.C.); shiue@ntuh.gov.tw (C.-Y.S.); 3 Department of Biology and Anatomy, National Defense Medical Center, Taipei 114, Taiwan; k6520319@yahoo.com.tw (Y.-T.J.); amys9520@gmail.com (L.-H.S.)

**Keywords:** Alzheimer’s disease (AD), bezafibrate, peroxisome proliferator-activated receptor (PPAR), tau pathology, cerebral glucose metabolism, neuroinflammation, positron emission tomography (PET)

## Abstract

Bezafibrate, a pan-peroxisome proliferator-activated receptor (PPAR) agonist, reportedly attenuated tau pathology in a transgenic mouse model of primary tauopathy. Since tau pathology is a neuropathological hallmark of Alzheimer’s disease (AD), bezafibrate may be a potential drug for the treatment of AD. However, no study has investigated its effects in AD models. Thus, we aimed to evaluate whether bezafibrate has neuroprotective effects in a sporadic AD model induced by streptozotocin (STZ) intracerebroventricular (ICV) injection. Rats were administered STZ-ICV (3 mg/kg) followed by bezafibrate (50 mg/kg/day, intraperitoneal) for 4 weeks. Behavior tests and positron emission tomography (PET) were performed to evaluate longitudinal changes in cognitive function, tau pathology, and cerebral glucose metabolism. Immunofluorescence staining was performed to assess neuronal survival and microglial accumulation. STZ-ICV administration induced significant cognitive impairment and substantial neuronal loss, tau pathology, glucose hypometabolism, and microgliosis in the cortex and hippocampus, while bezafibrate effectively attenuated these abnormalities. This study demonstrated that bezafibrate has long-lasting neuroprotective effects in a sporadic AD model. Our data indicate that the neuroprotective effects of bezafibrate might be associated with its ability to ameliorate tau pathology, brain glucose hypometabolism, and neuroinflammation. These findings suggest that bezafibrate is a potential multi-target drug candidate for the treatment of AD.

## 1. Introduction

Alzheimer’s disease (AD) is the most common cause of dementia and is characterized by progressive and irreversible neurodegenerative processes. Two neuropathological hallmarks of AD are deposition of beta-amyloid (Aβ) plaques and accumulation of hyperphosphorylated tau protein (p-tau) in the form of neurofibrillary tangles. Substantial evidence suggests that the accumulation of abnormally folded Aβ and tau pathology have a cause-effect relationship with neuronal degeneration and subsequent cognitive impairment; nevertheless, the precise pathogenesis of AD remains unclear [1]. Recent research suggests that AD might be a metabolic disease in which the brain is unable to efficiently utilize glucose for energy production due to brain insulin resistance [2,3]. In AD patients, brain insulin resistance can disrupt signaling pathways that modulate neuronal survival, accelerate the development of tau pathology, induce neuroinflammation, and eventually result in neurodegeneration [4,5]. Since brain insulin resistance and subsequent brain glucose hypometabolism, tau pathology, and neuroinflammation might occur simultaneously in AD patients, a therapeutic strategy that could collectively alleviate these unfavorable pathological changes will be useful in AD treatment.

Bezafibrate, a pan-peroxisome proliferator-activated receptor (PPAR) agonist used for the treatment of dyslipidemia for over 25 years with good safety, has been reported to improve peripheral glucose metabolism by activating PPARs and upregulating insulin sensitivity [6]. Bezafibrate can cross the blood-brain barrier (BBB) [7] and, thus, has the potential to improve cerebral insulin sensitivity and glucose metabolism in the same manner [8]. Furthermore, Dumont et al. demonstrated that bezafibrate treatment could attenuate the severity of tau pathology in a transgenic mouse model of primary tauopathy by improving energy metabolism, suppressing oxidative stress, and inhibiting neuroinflammation [9]. Taken together, bezafibrate might be a promising multi-target drug candidate that can simultaneously alleviate unfavorable pathological changes in AD patients. However, to the best of our knowledge, no study has investigated the neuroprotective effects of bezafibrate in animal models of sporadic AD, which accounts for more than 95% of all AD cases. Hence, we used a sporadic AD rat model induced by intracerebroventricular (ICV) injection of streptozotocin (STZ) to evaluate the effects of bezafibrate. This STZ-ICV-induced sporadic AD model expresses progressive Aβ and p-tau overaccumulation, accompanied by neuroinflammation, decreased brain glucose utilization, neuronal loss, and cognitive impairments. Therefore, it appears to be an excellent experimental model for the evaluation of sporadic AD-type neurodegeneration [10,11,12,13,14]. In the present study, the rats were intracerebroventricularly injected with STZ, followed by bezafibrate (50 mg/kg/day) intraperitoneal (IP) injection for 4 weeks. The short-term and long-term effects of bezafibrate on cognitive functions, tau pathology, and cerebral glucose metabolism were assessed using longitudinal animal behavior tests and positron emission tomography (PET), respectively, on the 4th, 8th, and 12th week after STZ-ICV administration. Immunofluorescence staining was performed at the third month to confirm the long-term protective effects of bezafibrate on neuronal survival, tau pathology, and microglial accumulation. We aimed to evaluate whether bezafibrate has neuroprotective effects on sporadic AD-type neurodegeneration.

## 2. Results

### 2.1. Bezafibrate Rescued the STZ-ICV-Induced Behavioral Deficits

**Figure 1:**
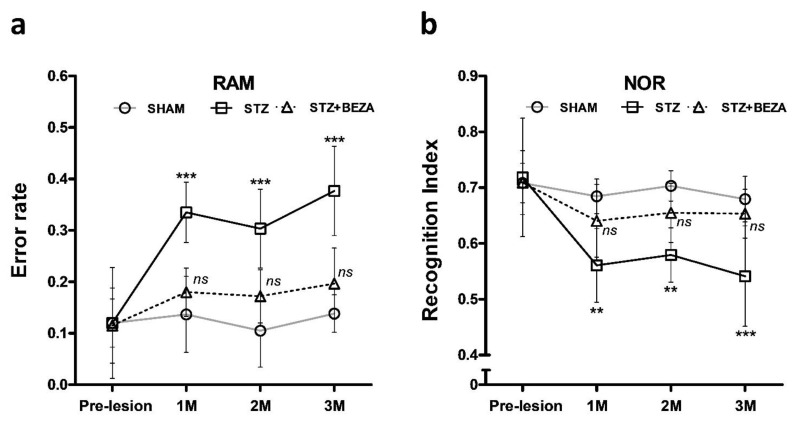
Bezafibrate treatment rescued the STZ-ICV-induced behavioral deficits. (**a**) RAM tests demonstrated that the error rates of the STZ group were significantly higher compared to those of the SHAM group after STZ-ICV administration. The STZ-ICV-induced abnormalities were successfully ameliorated by the 28-day bezafibrate treatment as there was no significant difference in error rates between STZ+BEZA and SHAM groups till the third month. (**b**) NOR tests revealed that the recognition indices in the STZ group were significantly decreased after STZ-ICV administration, while the recognition indices did not differ between STZ+BEZA and SHAM groups untill the third month. Data are presented as mean ± SD; **, *p* < 0.01, ***, *p* < 0.001, *ns*, not significant, vs. SHAM group on the same timepoint; *n* = 6, per group.

Radial arm maze (RAM) tests were performed at one-month intervals to assess the longitudinal change in spatial memory of animals after STZ-ICV administration along with the protective effects of bezafibrate (Figure 1a). In the SHAM group, the error rates did not show any significant differences after the ICV injection until the third month (*p* > 0.05, post- vs. pre-lesion). After STZ-ICV administration, error rates in the STZ group were significantly higher than those in the SHAM group (*p* < 0.001, STZ vs. SHAM). Following the 28-day bezafibrate treatment, there was only a slight but not statistically significant increase in the error rates of the STZ+BEZA group compared with those of the SHAM group (*p* > 0.05, STZ+BEZA vs. SHAM). It is noteworthy that the beneficial effects persisted until the third month, even when the treatment was stopped at the end of the first month.

Furthermore, we conducted novel object recognition (NOR) tests to evaluate the object recognition memory of animals (Figure 1b). Similar to the results of the RAM tests, the novel-object preference, expressed as “recognition index,” did not differ in the SHAM group after the ICV injection until the third month (*p* > 0.05, post- vs. pre-lesion). In contrast, the STZ group demonstrated significantly decreased recognition indices after STZ-ICV administration, compared to the SHAM group (*p* < 0.01 [1st, 2nd month] and <0.001 [3rd month], STZ vs. SHAM). In the STZ+BEZA group, the 28-day bezafibrate treatment also demonstrated a considerable and long-lasting protective effect on the object recognition memory, showing no significant difference in recognition indices between the STZ+BEZA and SHAM groups until the third month (*p* > 0.05, STZ+BEZA vs. SHAM). The results of both animal behavior tests suggest the potential protective effects of bezafibrate on cognitive function in the STZ-ICV-induced sporadic AD rat model.

### 2.2. Bezafibrate Mitigated the STZ-ICV-Induced Brain Neuronal Loss

**Figure 2:**
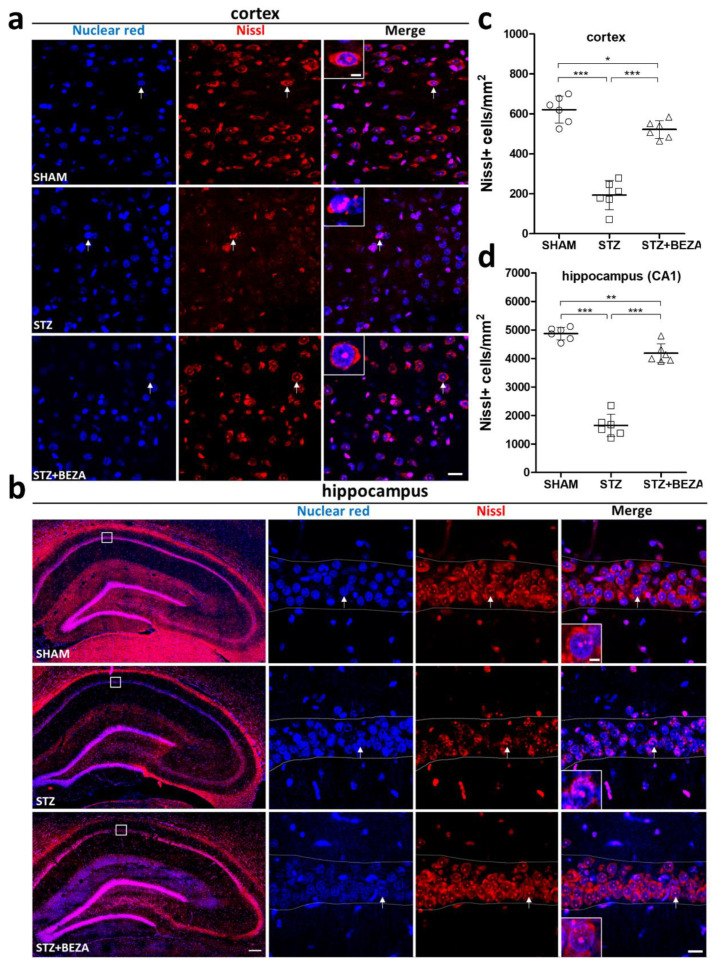
Bezafibrate mitigated the STZ-ICV-induced brain neuronal loss. Neuronal survival was evaluated by immunofluorescence staining with Nissl stain (red, for neurons) and nuclear red (blue, as nuclear counterstain). The Nissl staining-positive cells in the cortex (**a**) and hippocampus (**b**) of the STZ group (middle rows) were significantly fewer than those in the SHAM group (upper rows). The STZ+BEZA group (lower rows) demonstrated considerably preserved Nissl-positive cells in both the cortex and hippocampus. Quantitative analysis demonstrated that STZ-ICV administration caused a marked decrease in Nissl-positive cell densities in the cortex (**c**) and hippocampus (**d**) in the STZ group, while the reduction in density was remarkably alleviated by bezafibrate treatment in the STZ+BEZA group. Arrows indicate examples of Nissl-staining positive cells; dotted lines in the high-magnification images of panel b delineate the CA1 region of the hippocampus; scale bars, panel a: 20 µm, panel b: 200 µm in whole hippocampus images, 20 µm in high-magnification images, 5 µm in higher-magnification insets. Data are presented as mean ± SD; *, *p* < 0.05, **, *p* < 0.01, ***, *p* < 0.001; *n* = 6, per group.

To evaluate the protective effect of bezafibrate on STZ-ICV-induced neuronal injury, neuronal survival in the cortex and hippocampus was assessed by Nissl staining in the third month after STZ-ICV administration [15]. A significant loss of Nissl staining positive cells was observed in the cortex (Figure 2a,c) and hippocampus (Figure 2b,d) of the STZ group as compared to those in the SHAM group (cortex: 193.7 ± 29.5 vs. 621.2 ± 27.6 cells/mm^2^, CA1 region of the hippocampus: 1647.6 ± 161.9 vs. 4872.3 ± 90.3 cells/mm^2^, both *p* < 0.001, STZ vs. SHAM), suggesting that STZ administration induced significant neuronal injury in the brain. In the STZ+BEZA group, the STZ-ICV-induced loss of Nissl positive cells was significantly alleviated by bezafibrate treatment (cortex: 521.9 ± 18.3 cells/mm^2^, CA1 region of the hippocampus: 4180.4 ± 136.9 cells/mm^2^, both *p* < 0.001, STZ+BEZA vs. STZ), although the density of Nissl positive cells was slightly lower than that in the SHAM group (*p* < 0.05 [cortex] and <0.01 [hippocampus], STZ+BEZA vs. SHAM). These findings indicate that bezafibrate has beneficial effects on neuronal survival in the STZ-ICV-induced sporadic AD rat model.

### 2.3. Bezafibrate Alleviated the Severity of Tau Pathology in STZ-ICV-Injected Rats

**Figure 3:**
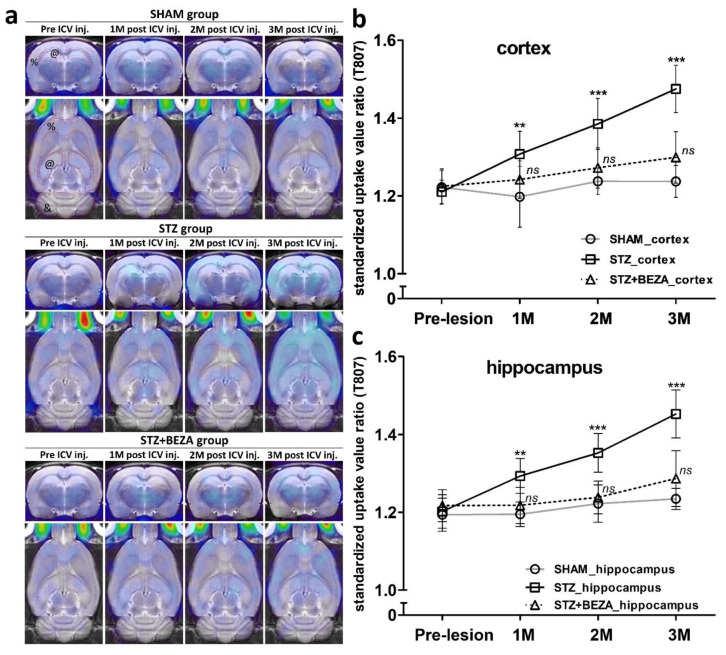
Bezafibrate attenuated PHF-tau accumulation in STZ-ICV-injected rats. Animal PET with T807 was used for the in-vivo measurement of PHF-tau accumulation. After STZ-ICV administration, the cerebral T807 uptake was gradually increased in the STZ group, but only minimally increased in the SHAM and STZ+BEZA groups (**a**). Quantitative analysis of T807 uptake revealed that STZ-ICV administration caused a progressive and significant increase in SUVRs in the cortex (**b**) and hippocampus (**c**) in the STZ group. Bezafibrate treatment successfully attenuated STZ-ICV-induced PHF-tau accumulation, as there was no significant difference in SUVRs between the STZ+BEZA and SHAM groups until the third month. Red dotted lines indicate representative delineation of cortex [%], hippocampus [@], and cerebellum [&]. Data are presented as mean ± SD; **, *p* < 0.01, ***, *p* < 0.001, *ns*, not significant, vs. SHAM group at the same time point; *n* = 6, per group.

Animal PET with [^18^F]T807 (T807) was used to assess the longitudinal change in paired helical filament-tau (PHF-tau) accumulation after STZ-ICV administration and to evaluate the protective effect of bezafibrate on tau pathology [16,17,18]. After STZ-ICV administration, the cerebral T807 uptake gradually increased in the STZ group, but only minimally increased in the SHAM and STZ+BEZA groups (Figure 3a). Quantitative analysis of the standardized uptake value ratios (SUVRs) of T807 uptake in the cortex (Figure 3b) and hippocampus (Figure 3c) revealed that the SUVRs in both brain regions were significantly higher in the STZ group than in the SHAM group after STZ-ICV administration, with an increasing tendency (*p* < 0.01 (1st month) and <0.001 (2nd, 3rd month0, STZ vs. SHAM). In contrast, the SUVRs in both brain regions did not differ statistically between the STZ+BEZA and SHAM groups until the third month, even though the bezafibrate treatment was only given in the first month (all *p* > 0.05, STZ+BEZA vs. SHAM). These findings imply that bezafibrate may have a long-lasting protective effect on STZ-ICV-induced tau pathology.

**Figure 4:**
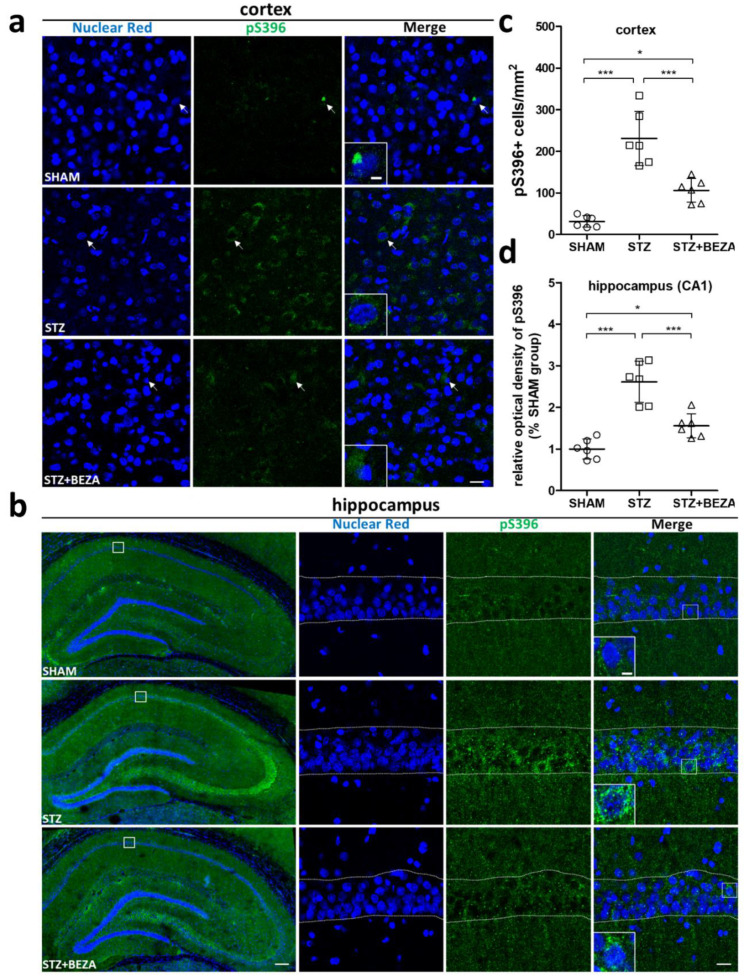
Bezafibrate reduced brain hyperphosphorylated tau protein accumulation in STZ-ICV-injected rats. Hyperphosphorylated tau protein was assessed by immunofluorescence staining with anti-pS396 antibody (green) and nuclear red (blue, as nuclear counterstain). The STZ group (middle rows) demonstrated a significant increase in pS396-positive cells in the cortex (**a**) as well as higher pS396 immunoreactivity in the hippocampus (**b**), compared to the SHAM group (upper rows). The STZ+BEZA group (lower rows) showed much lower pS396 immunoreactivity than the STZ group. Quantitative analysis revealed that STZ-ICV administration resulted in significantly higher pS396-positive cell densities in the cortex (**c**) and markedly increased relative optical densities of pS396 in the CA1 region of the hippocampus (**d**) in the STZ group, while bezafibrate treatment effectively attenuated the STZ-ICV-induced abnormalities in the STZ+BEZA group. Arrows in panel (**a**) indicate examples of pS396-staining positive cells; dotted lines in the high-magnification images of panel (**b**) delineate the CA1 region of the hippocampus; scale bars, panel (**a**): 20 µm, panel (**b**): 200 µm in whole hippocampus images, 20 µm in high-magnification images, 5 µm in higher-magnification insets. Data are presented as mean ± SD; *, *p* < 0.05, ***, *p* < 0.001; *n* = 6, per group).

To confirm the PET findings, immunofluorescence staining with anti-phospho-tau at serin 396 (pS396) antibody was conducted to assess the brain p-tau accumulation at the third month after STZ-ICV administration. Consistent with the T807 PET findings, the SHAM group only presented negligible pS396 positively-stained cells in the cortex, while the STZ group exhibited much higher pS396 positive cell density in the cortex (230.9 ± 26.8 vs. 31.2 ± 5.7 cells/mm^2^, *p* < 0.001, STZ vs. SHAM) (Figure 4a,c). In contrast to the STZ group, the STZ+BEZA group demonstrated a significantly lower density of pS396-positive cells in the cortex (106.1 ± 11.6 cells/mm^2^, *p* < 0.001, STZ+BEZA vs. STZ), although the density was still modestly higher than that of the SHAM group (*p* < 0.05, STZ+BEZA vs. SHAM). Similarly, the result of pS396 staining in the hippocampus was similar to the findings in the cortex (Figure 4b). Using the SHAM group as the standard, we found that the relative optical density (OD) of pS396 staining in the CA1 region of the hippocampus was significantly higher in the STZ group than in the SHAM group (2.61 ± 0.20, *p* < 0.001, STZ vs. SHAM), while bezafibrate treatment remarkably reduced the relative OD in the STZ+BEZA group (1.56 ± 0.12, *p* < 0.001, STZ+BEZA vs. STZ) (Figure 4d). The T807 PET and pS396 immunostaining findings collectively suggest that bezafibrate treatment could attenuate the severity of tau pathology in the STZ-ICV-induced sporadic AD rat model.

### 2.4. Bezafibrate Prevented STZ-ICV-Induced Cerebral Glucose Hypometabolism

**Figure 5:**
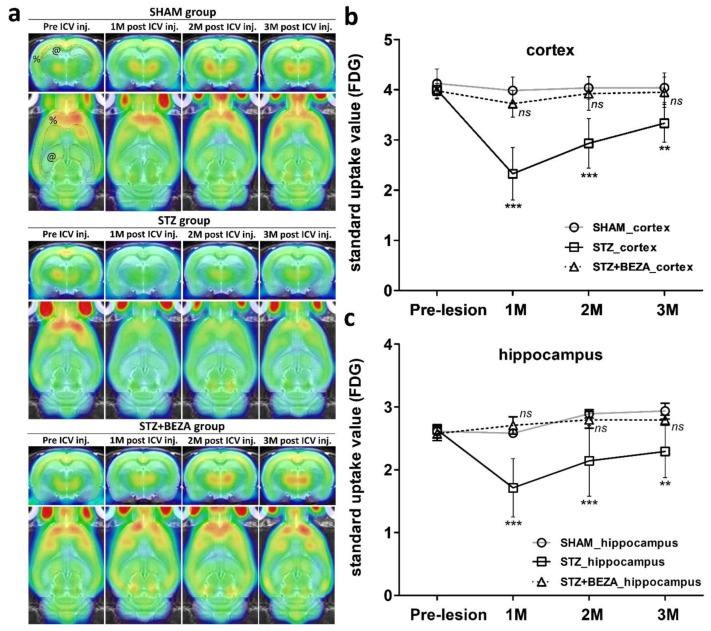
Bezafibrate prevented STZ-ICV-induced cerebral glucose hypometabolism. Animal PET with FDG was used for the in-vivo measurement of cerebral glucose utilization. After STZ-ICV administration, the cerebral FDG uptake was remarkably decreased in the STZ group but not in the SHAM and STZ+BEZA groups (**a**). Quantitative analysis of FDG uptake revealed that STZ-ICV administration caused a significant reduction in SUVs in the cortex (**b**) and hippocampus (**c**). The decline was most remarkable in the first month with a partial but incomplete recovery. Bezafibrate treatment successfully prevented STZ-ICV-induced cerebral glucose hypometabolism, as there was no significant difference in SUVs between the STZ+BEZA and SHAM groups until the third month. Red dotted lines indicate representative delineation of cortex [%] and hippocampus [@]. Data are presented as mean ± SD; **, *p* < 0.01, ***, *p* < 0.001, *ns*, not significant, vs. SHAM group at the same time point; *n* = 6, per group.

A previous study demonstrated that bezafibrate could improve peripheral glucose metabolism by regulating insulin sensitivity [6]. To evaluate the change in cerebral glucose utilization after STZ-ICV administration and to determine whether bezafibrate can improve cerebral glucose metabolism, [^18^F]fluorodeoxyglucose (FDG) PET was used for in-vivo measurement of longitudinal changes in brain glucose metabolism. After the ICV injection, the STZ group showed significantly decreased brain FDG uptake, while that in the STZ+BEZA and SHAM groups demonstrated little interval change (Figure 5a). Quantitative analysis of the standardized uptake values (SUVs) of FDG uptake in the cortex (Figure 5b) and hippocampus (Figure 5c) demonstrated that the SUVs in both brain regions in the STZ group were significantly lower than those in the SHAM group after STZ-ICV administration. The STZ-ICV-induced decline of SUVs was most remarkable in the first month and was gradually and partially recovered in the second and third months; however, the SUVs in the STZ group were still considerably lower than those in the SHAM group (*p* < 0.001 (1st and 2nd month) and <0.01 (3rd month), STZ vs. SHAM). In the STZ+BEZA group, there was no statistical difference in SUVs in the cortex and hippocampus compared to those in the SHAM group until the third month (all *p* > 0.05, STZ+BEZA vs. SHAM). These findings indicate that bezafibrate can rescue STZ-ICV-induced cerebral glucose hypometabolism.

### 2.5. Bezafibrate Reduced Microgliosis in STZ-ICV-Injected Rats

**Figure 6:**
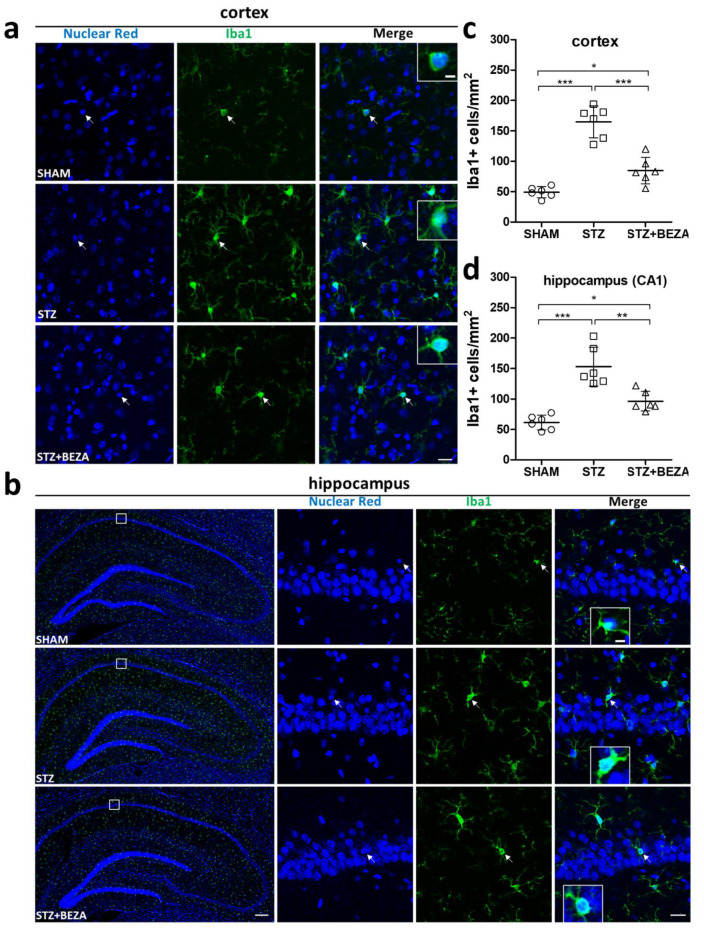
Bezafibrate reduced microgliosis in STZ-ICV-injected rats. Microgliosis was evaluated by immunofluorescence staining with anti-Iba1 antibody (green, for microglia) and nuclear red (blue, as nuclear counterstain). Iba1-staining positive cells were significantly increased in the cortex (**a**) and hippocampus (**b**) of the STZ group (middle rows), compared to those in the SHAM group (upper rows). The accumulation of Iba1-positive cells in both brain regions was less prominent in the STZ+BEZA group (lower rows) compared to that in the STZ group. Quantitative analysis demonstrated that STZ-ICV administration significantly increased densities of Iba1-positive cells in the cortex (**c**) and CA1 region of the hippocampus (**d**) in the STZ group, while bezafibrate treatment led to a considerably less density of Iba1-positive cells in both brain region in the STZ+BEZA group. Arrows indicate examples of Iba1-staining positive cells; scale bars, panel (**a**): 20 µm, panel (**b**): 200 µm in whole hippocampus images, 20 µm in high-magnification images, 5 µm in higher-magnification insets. Data are presented as mean ± SD; *, *p* < 0.05, **, *p* < 0.01, ***, *p* < 0.001; *n* = 6, per group.

Recent studies have demonstrated an emerging role for microglia in sporadic AD-type neurodegeneration [19,20,21]. Thus, we performed immunofluorescence staining with an anti-ionized calcium binding adaptor molecule 1 (Iba1) antibody to assess microglial accumulation in rat brains. Iba1-staining positive cells were significantly increased in the cortex (Figure 6a,c) and hippocampus (Figure 6b,d) of the STZ group, compared to those in the SHAM group (cortex: 164.8 ± 10.7 vs. 49.2 ± 3.7 cells/mm^2^, CA1 region of the hippocampus: 153.1 ± 13.0 vs. 61.5 ± 4.9 cells/mm^2^, both *p* < 0.001, STZ vs. SHAM). In the STZ+BEZA group, the STZ-ICV-induced overaccumulation of Iba1-positive cells was reduced by bezafibrate treatment in both brain regions (cortex: 84.9 ± 8.9 cells/mm^2^, CA1 region of the hippocampus: 96.5 ± 6.6 cells/mm^2^, *p* < 0.001 and <0.01, STZ+BEZA vs. STZ), although it was still slightly more prominent than that in the SHAM group (both *p* < 0.05, STZ+BEZA vs. SHAM). These findings indicate that bezafibrate could partially inhibit STZ-ICV-induced microgliosis in both the cortex and hippocampus.

## 3. Discussion

The present study evaluated the potential protective effects of bezafibrate on sporadic AD-type neurodegeneration. We observed that STZ-ICV administration induced sporadic AD-like changes, including significant cognitive impairment as well as substantial neuronal loss, tau pathology, glucose hypometabolism, and microgliosis in the cortex and hippocampus of rats. Bezafibrate treatment for 28 days effectively attenuated these abnormalities and normalized the condition towards the SHAM control group. These findings provide a mechanistic basis for the protective effects of bezafibrate on sporadic AD-type neurodegeneration.

In our study, animal behavior tests were conducted to assess longitudinal changes in cognitive function after STZ-ICV administration. We observed significant cognitive impairment in the first month after STZ-ICV administration, as shown by the considerably increased error rates and decreased recognition indices in RAM and NOR tests, respectively, which was aligned with previous studies [13,22]. The behavioral deficits noted in the RAM and NOR tests may indicate damages in spatial and object-recognition memory, respectively, which have been widely reported to be compromised in AD development [23,24]. Treatment with bezafibrate for 28 days successfully rescued the STZ-ICV-induced cognitive impairment, while the protective effect persisted until the third month. To the best of our knowledge, there are no available data on the effects of bezafibrate on cognitive function. Our study is the first to show that bezafibrate treatment could prevent cognitive impairment in a rat model of sporadic AD. Furthermore, impairments in spatial and object recognition memory have been previously associated with cortical and hippocampal damage [23,25,26]. Significant neuronal loss was observed in the cortex and hippocampus of STZ-ICV-injected rats, which might explain the cognitive impairment demonstrated in the behavioral tests. Similar to the protective effect on cognition, bezafibrate treatment considerably attenuated STZ-ICV-induced neuronal loss in both brain regions, although not fully rescued. Together, these findings support that bezafibrate has a neuroprotective effect in a sporadic AD model.

In addition to cognitive function and neuronal survival, we also investigated tau pathology to better understand the possible mechanisms underlying the neuroprotective effect of bezafibrate in a sporadic AD model. The development of tau pathology in sporadic AD is a progressive process [27], in which the soluble monomers of tau protein are hyperphosphorylated and pathologically aggregated to form PHF-tau, which, in turn, assembles to generate neurofibrillary tangles, leading to subsequent neuronal injury and cognitive decline [28]. T807 PET was applied in our research for the longitudinal evaluation of tau pathology to comprehensively assess the therapeutic effect of bezafibrate. The advantage of PET is that it is an in-vivo imaging tool that allows noninvasive monitoring of biological and pathological processes at the molecular level [29,30]. PET with T807 (a tau-specific radiotracer) has been broadly applied in both preclinical and clinical studies to evaluate the severity of tau pathology and the progression of AD [16,17,18]. The results obtained in our study show a time-dependent increase in T807 uptake in the cortex and hippocampus of the STZ group after the first month when compared with the SHAM group, indicating progressive development of tau pathology after STZ-ICV administration. Treatment with bezafibrate for 28 days successfully attenuated the STZ-ICV-induced tau pathology in the first month, while the benefits lasted until the third month. Immunofluorescence staining with anti-pS396 antibody at the third month further confirmed significantly increased p-tau immunoreactivity in these brain regions, while bezafibrate treatment effectively reduced it, indicating that bezafibrate has a long-lasting protective effect on tau pathology in STZ-ICV-injected rats. It is possible that the protective effect of bezafibrate on tau pathology further contributes to its neuroprotective effect. The tau pathology observed after STZ-ICV administration will undoubtedly lead to subsequent neuronal injury [1]; however, the damaged neurons release more p-tau or PHF-tau into the extracellular space. These pathological tau proteins can be transported to connected neurons via exosomes or ectosomes, resulting in misfolding and formation of toxic tau pathology [31]. The recipient neurons may further spread the pathological tau fragments to other neurons, which induces neuron-to-neuron prion-like propagation of tau pathology and eventually forms a vicious cycle that exacerbates neurodegeneration [27]. Hence, it can be speculated that the neuroprotective effect of bezafibrate observed in our study may be partly achieved by attenuating tau pathology to break this vicious circle.

Apart from tau pathology, impaired cerebral glucose utilization was also observed after STZ-ICV administration, confirmed by a significant decrease in FDG uptake in the cortex and hippocampus. Bezafibrate treatment successfully rescued the STZ-ICV-induced brain glucose hypometabolism, similar to the protective effects on tau pathology observed in our study. These findings suggest an association between cerebral glucose hypometabolism and the development of tau pathology. Sporadic AD has been referred to as “type 3 diabetes” by some researchers [2,32,33,34]. Recent studies focused on insulin resistance and subsequent brain glucose hypometabolism provide a new perspective on the molecular mechanisms that may contribute to the pathogenesis of tau pathology in AD [3,4,5,10]. First, tau protein has approximately 15 serine/threonine residues that are canonical sites for proline-directed protein kinases such as glycogen synthase kinse-3β (GSK-3β). GSK-3β is negatively regulated via the phosphoinositide 3-kinase-Akt (PI3K-Akt) signaling pathway. Second, the PI3K-Akt signaling pathway is activated by the insulin receptor, which is downregulated in insulin resistance. Collectively, insulin resistance induces hyperactivity of GSK-3β, which leads to tau hyperphosphorylation and subsequent PHF-tau and neurofibrillary tangle formation [5]. In addition, insulin resistance and dysregulation of insulin signaling in AD brains also cause lower expression of glucose transporter (GLUT), thus impairing glucose uptake in the neurons. Decreased glucose uptake/metabolism leads to a lower level of uridine diphosphate *N*-acetylglucosamine (UDP-GlcNAc), a product of a branch of glucose metabolic pathway, and further leads to decreased O-GlcNAcylation of tau protein. Since O-GlcNAcylation regulates tau phosphorylation inversely, its downregulation might facilitate hyperphosphorylation of tau protein [35,36]. In summary, insulin resistance and glucose hypometabolism in AD brains may provoke tau hyperphosphorylation and subsequent tau pathology.

Considering these points and the simultaneous development of tau pathology and brain glucose hypometabolism in the STZ-ICV sporadic AD model, it is reasonable to assume that brain insulin resistance and glucose hypometabolism may play an important role in the development of tau pathology in our model. A previous study that used the same STZ-ICV sporadic AD rat model demonstrated that STZ-ICV administration caused a decrease in PI3K-Akt signaling activity, GLUT expression, tau O-GlcNAcylation, hyperactivity of GSK-3β, and subsequent tau hyperphosphorylation [10]. In another study, Chen et al. applied FDG PET to evaluate brain glucose utilization in a unilateral STZ-ICV rat model and found that FDG uptake in the cortex and hippocampus of the injected side was significantly decreased in the second month after STZ-ICV administration [37], which is similar to our findings. In our study, we evaluated not only one time point but also the longitudinal change in brain glucose metabolism until the third month, and we found significant cerebral glucose hypometabolism in the first month after STZ-ICV administration. Surprisingly, the hypometabolism demonstrated a spontaneous and partial recovery, which might be explained by the fact that STZ-ICV injection causes an intensive acute response in the first month and could be partly compensated for until the third month [11]. Moreover, we found that bezafibrate treatment for 28 days effectively prevented STZ-ICV-induced glucose hypometabolism from the first month until the third month, which indicates a long-lasting protective effect of bezafibrate on cerebral glucose metabolism. Although no previous research has specifically investigated the effect of bezafibrate on cerebral glucose metabolism, several studies have demonstrated that bezafibrate could improve peripheral insulin sensitivity and glucose utilization in diabetic mouse models or patients [6,38,39]. Data from animal studies suggest that the underlying molecular mechanisms might be attributed to the activation of PPARs, which improves metabolic flexibility, insulin sensitivity, and glucose metabolism [6,38]. PPARs are a family of ligand-regulated nuclear receptors. They have been shown to play essential roles in energy metabolism, insulin sensitization, and inflammation [40]. In recent years, an increasing number of studies have emphasized the role of PPARs and their ligands as ideal candidates for the treatment of AD [8,40,41,42,43]. With regard to the fact that all three PPAR isoforms (PPAR-α, PPAR-β/δ, and PPAR-γ) are expressed in the brain [32] and that bezafibrate can cross the BBB [7] and activate all three PPAR isoforms at comparable doses [44], it could conceivably be hypothesized that the effect of bezafibrate on cerebral glucose utilization might be achieved via PPARs activation. However, further research should be conducted to investigate the effect of bezafibrate on different PPAR isoforms in sporadic AD.

In addition to cerebral glucose hypometabolism, growing evidence supports the important role of neuroinflammation in the pathogenesis of AD [45,46]. Neuroinflammation, as reflected by microgliosis and other reactive gliosis, has been widely observed in a variety of neurodegenerative diseases, including AD [19,47,48]. Recent research has also shown that microglia can modulate neuronal loss in AD [20,21]. In our study, STZ-ICV administration caused substantially increased microglia accumulation in the cortex and hippocampus, which is in line with previous research [49,50]. Meanwhile, the STZ-ICV-induced microgliosis was partly attenuated by bezafibrate treatment, implying that bezafibrate has a beneficial effect on neuroinflammation. Activation of PPARs not only improves glucose metabolism, but also mitigates neuroinflammation. The anti-neuroinflammatory effects of PPARs in AD are well established. Activation of PPARs in any of the three isoforms is known to inhibit microglial activation and mitigate neuroinflammation [42,43,51,52,53]. Since bezafibrate is a pan-PPAR agonist, it can be assumed that the neuroprotective effect of bezafibrate may be partly achieved through the inhibition of neuroinflammation. However, further research is required to clarify this issue.

Our study has successfully demonstrated that bezafibrate can ameliorate tau pathology, brain glucose hypometabolism, and neuroinflammation, as well as exert neuroprotective effects, in a sporadic AD model. However, a limitation of this study is that since only a small amount of Aβ is detectable in the brains of STZ-ICV rats within 3 months after STZ-ICV administration [11], the effect of bezafibrate on amyloid genesis was not investigated. Currently, data regarding the effect of bezafibrate on the amyloidogenic pathway of AD are not available. Nevertheless, previous studies have shown a relationship between insulin resistance and amyloid genesis in AD [3]. For example, while insulin-degrading enzyme (IDE) degrades insulin, it can also degrade extracellular Aβ and eliminate Aβ-associated neurotoxic effects. Under circumstances of insulin resistance, the expression of IDE is decreased due to downregulation of the PI3K-Akt signaling pathway [4]. Taken together, insulin resistance could decrease the expression of IDE and exacerbated Aβ aggregation. As our data suggest that bezafibrate has the potential to improve cerebral insulin sensitivity and glucose utilization, it is reasonable to speculate that bezafibrate can also improve amyloid genesis by enhancing the insulin signaling pathway. Therefore, further studies with a longer follow-up time should be performed to assess the effects of bezafibrate on amyloid genesis in the STZ-ICV-induced sporadic AD rat model.

## 4. Materials and Methods

### 4.1. Animals

Adult male Sprague–Dawley rats (280–300 g, 8 weeks of age), obtained from the BioLASCO Taiwan Co., Ltd. (Taipei, Taiwan), were housed in the Laboratory Animal Center of National Defense Medical Center (NDMC, Taipei, Taiwan), which has been fully accredited by the Association for Assessment and Accreditation of Laboratory Animal Care International (AAALAC) since 2007. The rats were subjected to a 12-h light/dark cycle with ad libitum access to food and water at a constant temperature of 23 ± 2 °C. The executed animal experiment protocols were approved by the Institutional Animal Care and Use Committee of NDMC (certification number: IACUC-19-135).

### 4.2. Animal Groups and Drugs Treatments

#### 4.2.1. Animal Group Assignments and Experimental Procedures

**Figure 7:**
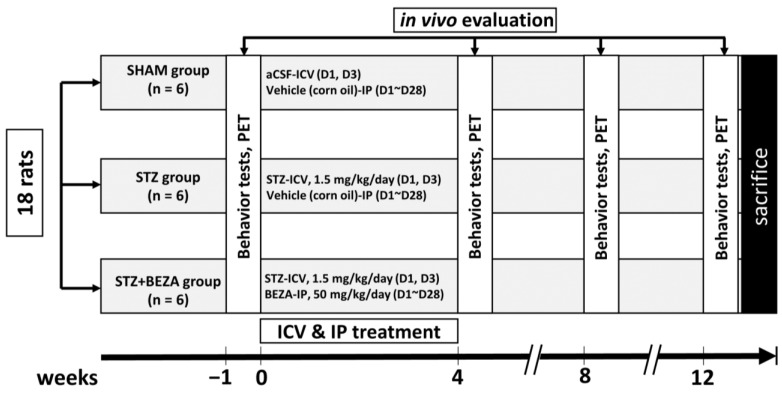
Diagram of group assignments and experimental procedures. Rats were divided into three groups: SHAM, STZ, and STZ+BEZA. In-vivo evaluation (behavior tests and PET) was performed before and 4, 8, and 12 weeks after ICV injection.

Animals were randomly divided into three groups: STZ-ICV-injected rats (STZ group, *n* = 6), rats that received STZ-ICV injection followed by bezafibrate intraperitoneal injection for 28 days (STZ+BEZA group, *n* = 6), and sham-operated rats that underwent ICV injection with artificial cerebrospinal fluid (aCSF) (SHAM group, *n* = 6). Behavior tests and animal PET were performed in each group before and at 1, 2, and 3 months after the ICV injection for in-vivo evaluation. Rats were sacrificed after all studies were completed, and brains were rapidly excised for immunofluorescence staining. The group assignments and experimental procedures are shown in Figure 7.

#### 4.2.2. Procedures of STZ-ICV Administration and Bezafibrate Treatment

Previously described procedures of ICV administration of STZ were implemented with minor modifications [37,54]. Briefly, rats were deeply anesthetized and restrained in a stereotaxic apparatus (Stoelting, Wood Dale, IL, USA). STZ (Sigma–Aldrich, Saint Louis, MO, USA) was freshly dissolved in aCSF, and a solution of 25 mg/mL was prepared. ICV injection was performed bilaterally with STZ (3 mg/kg) in two divided doses, on days 1 and 3 (i.e., 0.75 mg/kg into each lateral ventricle per day) or with the same volume of aCSF. The coordinates were 0.8 mm posterior to the bregma, 1.5 mm bilateral to the sagittal suture, and 3.6 mm below the dura. Bezafibrate powder (Sigma–Aldrich) was dissolved in sterile corn oil at 40 °C to obtain a suspension with a final concentration of 50 mg/mL. Bezafibrate or vehicle (corn oil) was intraperitoneally injected once a day from days 1 to 28. The dose of bezafibrate IP injection was 50 mg/kg/day, which has been used to reduce the inflammatory response in rats [55].

### 4.3. Animal Behavior Tests

#### 4.3.1. Radial Arm Maze Test

The RAM test was modified as previously described and used to test the spatial memory of animals [56]. The maze has eight arms, numbered from 1 to 8 (60 cm × 10 cm), with an extension radially from the central area of 30 cm in diameter. A food reinforcer (50 mg food pellet) was placed at the end of each arm. During the training (on days 1 and 2, one trial per day), each rat was individually placed in the maze and given a maximum of 10 min to visit all eight arms for the food reinforcer. Test trials were performed on days 3 and 4 with the same settings. Errors were recorded as reentries into the arms previously visited within the same trial. The error rate was calculated as “(number of errors/total entries) × 100%”.

#### 4.3.2. Novel Object Recognition Test

In the NOR tests, the novel-object preference of rats was observed to assess the object-recognition memory. When subjects are exposed to a novel and familiar object simultaneously, they have a propensity to spend more time investigating the novel object. The loss of preference for novelty may indicate alterations in cognitive function [26]. The apparatus consisted of a black acrylic open field box (48 cm × 48 cm × 48 cm). This behavioral test has three phases: habituation, familiarization, and testing. In the habituation phase (day 1), rats were placed into the open-field arena to freely explore the environment, in the absence of an object for approximately 10 min. In the familiarization phase (day 2), the rats explored the open field with two identical objects positioned opposite at a distance of 9 cm from the walls for 10 min. On the third day of the testing phase, each rat was allowed to explore for 5 min to be acquainted with these two identical objects. Next, one of the two familiar objects is substituted with a novel object. After a 15 min interval, the rat returned to the same arena for another 5 min test run. The recognition index was calculated using the formula “TN/(TN + TF) × 100%”, where TN and TF are the time spent exploring the novel and familiar objects, respectively [57].

### 4.4. Animal PET and Radiopharmaceuticals

Animal PET was performed using a small animal PET scanner (BIOPET 105, BIOSCAN, Santa Clara, CA, USA) with T807 and FDG. T807 has a high binding affinity to PHF-tau (aggregated from p-tau) and can be used to assess the severity of tau pathology [16]. FDG is a glucose analog that can be used to measure glucose utilization in the brain [58]. Both radiopharmaceuticals were synthesized and provided by the Department of Nuclear Medicine affiliated with the Tri-Service General Hospital.

The protocol for small-animal PET imaging was modified from a previous study [12,59,60]. Briefly, rats were fasted for at least 8 h prior to the PET image acquisition. Before T807 administration, cyclosporin A (25 mg/kg; Sigma–Aldrich) was slowly administered through the tail vein to facilitate T807 passage through the BBB [61,62]. T807 (29.6–44.4 MBq; 0.8–1.2 mCi) or FDG (66.6–81.4 MBq; 1.8–2.2 mCi) is injected via the tail vein. After an uptake period (5 min for T807 and 45 min for FDG), static data acquisition was acquired (20 min for T807 and 30 min for FDG). AMIDE software 1.0.4 (Stanford University, Palo Alto, CA, USA) was used for PET data analysis. The rat brain atlas and magnetic resonance imaging results were applied for brain region confirmation and volume of interest (VOI) delineation [63]. VOIs of the cortex, hippocampus, and cerebellum on the PET images were delineated for subsequent quantitative analysis. FDG uptake values in the cortex and hippocampus were reported as SUVs, which represent the mean radioactivity for each VOI, normalized to the injected dose per body weight of each rat. In T807 PET, because the uptake was much lower in cortex and hippocampus (vs. FDG) and there was no marked interval change in cerebellar uptake after the STZ-ICV injection, the SUVR (calculated as “SUV_target brain region_/SUV_cerebellum_”) was applied to better represent the uptake in each individual brain region [64].

### 4.5. Immunofluorescence Staining

Rats were deeply anesthetized and transcardially perfused with 0.9% saline followed by 4% paraformaldehyde in 0.1 M phosphate-buffered saline (PBS). The brains were post-fixed and cryoprotected using sucrose solutions. Brains were sliced into a series of 30-μm coronal sections on a cryostat (CM3050, Leica Microsystems, Wetzlar, Germany). All brain slices (approximately 90 slices per animal) that contained the hippocampus and surrounding cortex were divided into three sets for Nissl staining and immunofluorescence staining with pS396 (a common form of phosphorylated tau protein in the AD brain [65]) and Iba1 (a microglia/macrophage-specific calcium-binding protein) antibodies. Nissl staining was performed to assess neuronal survival [15]. pS396 staining was performed to evaluate p-tau accumulation [66]. Iba1 staining was used to determine the severity of microgliosis and to estimate neuroinflammation [19,21]. Brain slices were stained with Nissl stain (1:150 dilution; N-21482; Invitrogen, Waltham, MA, USA), anti-pS396 antibody (1:500 dilution; ab109390; Abcam, Cambridge, UK), or anti-Iba1 antibody (1:500 dilution; 019-19741; Wako, Tokyo, Osaka, Japan). For pS396 and Iba1 staining, the slices were stained with Alexa Fluor 488-conjugated donkey anti-rabbit IgG (1:250 dilution; Jackson ImmunoResearch Laboratories, West Grove, PA, USA). Finally, the brain slices were stained with nuclear red (1:1000 dilution; AAT Bioquest, Inc., Sunnyvale, CA, USA) for nuclear quantitation. The fluorescence images of each section were captured using a confocal microscope (LSM880, Zeiss, Oberkochen, Germany). Three sections were analyzed per animal per staining to obtain one result for each rat. Results are expressed as the average number of Nissl, pS396 and Iba1-staining positive cells in the cortex and hippocampus (cells per mm^2^), except for the quantification of pS396 in the hippocampus. Since pS396 staining in the hippocampus was more prominent in neurites than in neuronal bodies, as previously described [10], the OD of pS396 was used instead of the cell count to semi-quantify the abundance of p-tau accumulation in the hippocampus. pS396 OD measurements of the CA regions of the hippocampus were quantified based on the intensity of the green channel on RGB images with background correction (normalized by corpus callosum) with Image-Pro Plus 6.0 (Media Cybernetics, Rockville, MD, USA). The results are expressed as relative OD, calculated as “OD _treated group_/OD _SHAM group_” [67].

### 4.6. Statistical Analysis

Statistical analysis of the data obtained from the behavioral tests, PET, and immunofluorescence staining of all experimental groups was performed using GraphPad Prism software version 5.01 (GraphPad Software, San Diego, CA, USA). Data are expressed as mean ± standard deviation. The statistical significance of differences between the experimental groups was determined using two-way ANOVA (for the parameters in behavioral tests, and SUVs and SUVRs in PET) or one-way ANOVA (cell counts and relative ODs in immunofluorescence staining), followed by post-hoc Bonferroni test. Statistical significance was set at *p* < 0.05.

## 5. Conclusions

Our study has shown that bezafibrate exerts considerable and long-lasting protective effects on cognitive impairment, neuronal loss, tau pathology, cerebral glucose hypometabolism, and neuroinflammation induced by STZ-ICV administration. To our knowledge, this is the first in-vivo study to evaluate the neuroprotective benefits of bezafibrate, a known pan-PPAR agonist, in a sporadic AD animal model. Our investigation provides a mechanistic basis for the neuroprotective effects of bezafibrate that may be associated with its ability to attenuate tau pathology, improve brain glucose metabolism, and reduce neuroinflammation, which might be achieved via activation of PPARs. The findings of this study suggest that bezafibrate is a potential multi-target drug candidate for the treatment of AD.

## Data Availability

The data presented in this study are available on request from the corresponding author.
